# Long-Term Hypoxia Upregulates Wnt and TGFβ1 Signaling in Eccrine Sweat Gland Cells In Vitro

**DOI:** 10.3390/ijms26146664

**Published:** 2025-07-11

**Authors:** Yanlin Lyu, Hiroko Kato, Qianwen Luo, Naoya Otani, Tateki Kubo, Kiyotoshi Sekiguchi, Fumitaka Fujita

**Affiliations:** 1 Laboratory of Advanced Cosmetic Science, Graduate School of Pharmaceutical Sciences, The University of Osaka, Yamada-oka, Suita 565-0871, Osaka, Japan; 2 The Department of Plastic Surgery, Graduate School of Medicine, The University of Osaka, Yamada-oka, Suita 565-0871, Osaka, Japan; 3 Division for Matrixome Research and Application, Institute for Protein Research, The University of Osaka, Yamada-oka, Suita 565-0871, Osaka, Japan; 4 Mandom Corporation, Chuo-Ku 540-8530, Osaka, Japan

**Keywords:** sweat gland, hypoxia, Wnt signaling, TGFβ1 signaling, stem cell

## Abstract

Eccrine sweat glands play a vital role in human thermoregulation; however, their self-repair function is minimal. Therefore, developing methods to regenerate and improve sweat gland function that use cultured sweat gland cells presents an urgent issue. The tissue microenvironment, especially hypoxic niches, essentially maintain cell stemness, highlighting the importance of oxygen concentration in the culture environment. Therefore, we evaluated the effects of different oxygen environments on human sweat glands and their regulatory mechanisms. Human eccrine sweat glands express HIF-1α and HIF-2α, suggesting that they respond to hypoxia in vivo. Primary human-derived eccrine sweat gland cells were cultured for two weeks using the spheroid culture method at 0.5%, 2%, 10%, and 21% O_2_ concentration. HIF-1, Wnt/β-Catenin, and TGFβ1 signaling increased in sweat gland cells cultured in 0.5% O_2_ conditions, along with increased undifferentiated cell marker expression. The results of this study will contribute to in vitro research models of sweat glands and treatment development for damage to sweat glands, including burns.

## 1. Introduction

Sweat glands crucially contribute to regulating body temperature [1]. Sweat glands are classified into eccrine and apocrine glands [1]. Apocrine glands are localized in specific areas, such as the axilla, and secrete odoriferous sweat, which is a pheromone remnant. In contrast, eccrine glands are distributed almost throughout the human body and secrete near-odorless sweat in response to temperature and psychogenesis [1]. With an increase in patients suffering from heat stroke and a predicted increase due to rising temperatures, countermeasures are urgently required [2]. Increasing perspiration from eccrine sweat glands (ESGs) is important in preventing heat stroke [1]. Medical interventions to improve sweating to prevent heat stroke have not been actively implemented for groups at risk of heat stroke caused by aging-related sweating disorders or other diseases [3]. In addition, patients with congenital anomalies, burns, trauma, and anhidrosis are at-risk groups for heat stroke because such afflictions are associated with sweating disorders. However, many unresolved pathogeneses and difficult-to-treat cases require an improved understanding of the disease and effective treatment methods [1]. In contrast, patients with hyperhidrosis, who perspire profusely to the extent that it interferes with daily life, have a significantly reduced quality of life [1]. Therefore, understanding and evaluating the sweating function and improving sweat gland function through drug discovery, transplantation therapy, and healthcare products are highly necessary.

Murine sweat glands are found only in the palmoplantar region and mainly serve a slip prevention function rather than a temperature regulation function. Moreover, there is no distinction between clear and dark cells in the secretory cells, and marker expressions are different [4,5], indicating that murine sweat glands have a different function than those of humans. Therefore, an in vitro human ESG model is needed. ESGs comprise secretory and excretory ducts [6]. The secretory duct is composed of αSMA (α-smooth muscle actin)-positive myoepithelial cells in the outer layer and K8 (Keratin8)-positive luminal cells in the inner layer [6], whereas the excretory duct comprises S100A2 (S100 calcium-binding protein A2)-positive basal layer cells in the outer layer and S100P (S100 calcium-binding protein P)-positive luminal cells. Myoepithelial cells include stem cells that can self-renew [6]. The ability of stem cells to maintain self-renewal and differentiate into specific cell lineages in cell culture is crucial for regenerative medicine applications, disease modeling, and drug discovery [7,8]. Cell culture is affected by endogenous and exogenous signals [9]; changes in the microenvironment of the stem cell niche, particularly in oxygen (O_2_) levels, play an important role in regulating their biological behavior [9]. Atmospheric O_2_ levels (21% O_2_/21 kPa/160 mmHg) are widely used in traditional cell culture, whereas in vivo O_2_ levels are noticeably lower at the tissue level [10]. When stem cells are cultured under conditions that differ from the O_2_ concentrations of their in vivo niche, they undergo a series of biological changes, including increased oxidative stress, altered metabolic turnover, decreased self-renewal, impaired motility, altered differentiation potential, and even loss of stem cell maintenance [11]. However, culturing stem cells in an environment compatible with their physiological oxygenation levels can inhibit these effects and maintain their native functions [11]. Therefore, a hypoxic culture environment enables long-term culturing of sweat gland cells in vitro while maintaining their stem cell nature. Physiological O_2_ concentrations in multiple tissues, varying between 1–14%, have been measured by various methods [12]. For example, physiological O_2_ concentrations in major organs, such as the lungs, heart, liver, and cerebrum, are lower than the atmospheric O_2_ concentration of 21% [13]. For skin tissue, physiological pO_2_ in the dermis is 76 mmHg, with the epidermis being moderately hypoxic (3.8–76 mmHg). Hair follicles and sebaceous glands are mildly-to-severely hypoxic (0.76–19 mmHg). Hypoxia-inducible factor 1 alpha (HIF-1α) expression has also been reported in sweat glands, suggesting that sweat glands respond to a hypoxic environment in vivo [14,15].

Accordingly, we hypothesized that reproducing a hypoxic microenvironment in sweat gland culture will enable the regulation of sweat gland stemness and differentiation. This study aimed to elucidate the effects of hypoxic environments on human sweat glands and their regulatory mechanisms. Furthermore, by analyzing the hypoxic response of sweat glands’ constituent cells, we elucidated the molecular mechanisms involved in maintaining and differentiating sweat gland stem cells. This study’s findings will contribute to establishing in vitro human sweat gland models and promote their application in regenerative medicine.

## 2. Results

### 2.1. Human Sweat Glands and Primary Cultured Sweat Gland Cells Express HIF-1α and HIF-2α Under Hypoxia

Hypoxia-inducible factors (HIFs) are the main transcription factors that enable cells to react to hypoxia. Immunohistochemical staining confirmed HIF-1α and HIF-2α expression in human skin tissue.

**Figure 1:**
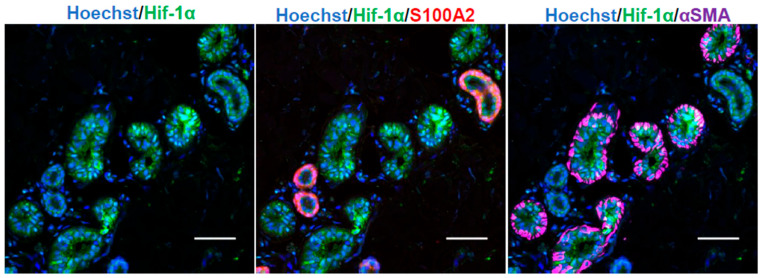
Expression of HIF-1α in sweat glands of human skin tissue. Blue indicates nuclei in Hoechst; green indicates HIF-1α; red indicates S100A2, a marker for excretory basement layer cells; and magenta indicates αSMA, a marker for myoepithelial cells (*n* = 3). Scale bar: 50 µm. Original magnification ×40.

**Figure 2:**
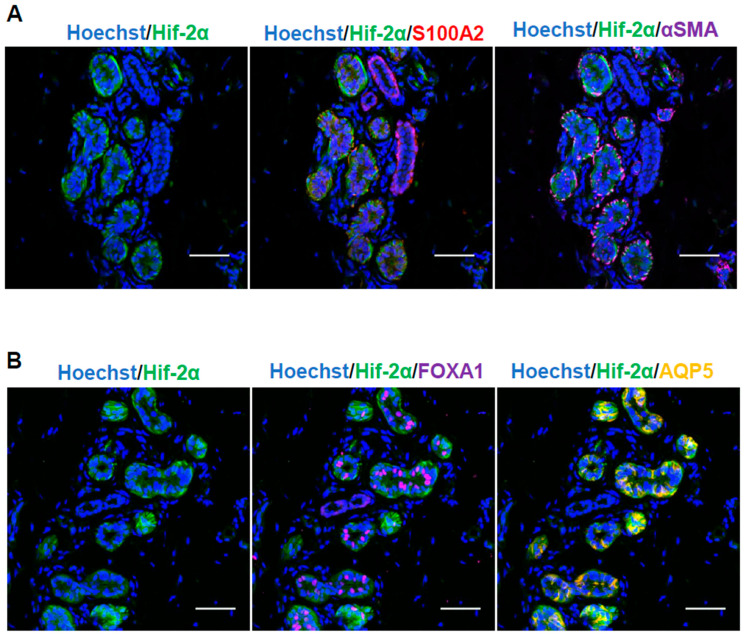
Expression of HIF-2α in sweat glands of human skin tissue. (**A**) Expression of HIF-2α in the secretory ducts and excretory ducts. Blue indicates nuclei in Hoechst; green indicates HIF-2α; red indicates S100A2, a marker for conduit basal layer cells; and magenta indicates αSMA, a marker for myoepithelial cells (*n* = 3). Scale bar: 50 µm. Original magnification ×40. (**B**) HIF-2α expression in the secretory ducts. Blue indicates nuclei in Hoechst; green indicates HIF-1α; magenta indicates FOXA1, a marker of dark cells; and orange indicates AQP5, a marker of clear cells (*n* = 3). Scale bar: 50 µm. Original magnification ×40.

HIF-1α was expressed in both the secretory ducts, which were surrounded by αSMA-positive myoepithelial cells in the ESGs, as well as the excretory ducts, where S100A2-positive cells were located on the outer side (Figure 1). Conversely, HIF-2α was localized only in the secretory area (Figure 2A).

Furthermore, in the secretory duct, HIF-2α was explicitly expressed in myoepithelial (Figure 2A) and AQP5-positive clear cells (Figure 2B), whereas expression was not observed in FOXA1-positive dark cells (Figure 2B). This finding suggests that HIF-1α and HIF-2α may be involved in regulating sweat gland physiological activity in human skin tissue.

**Figure 3:**
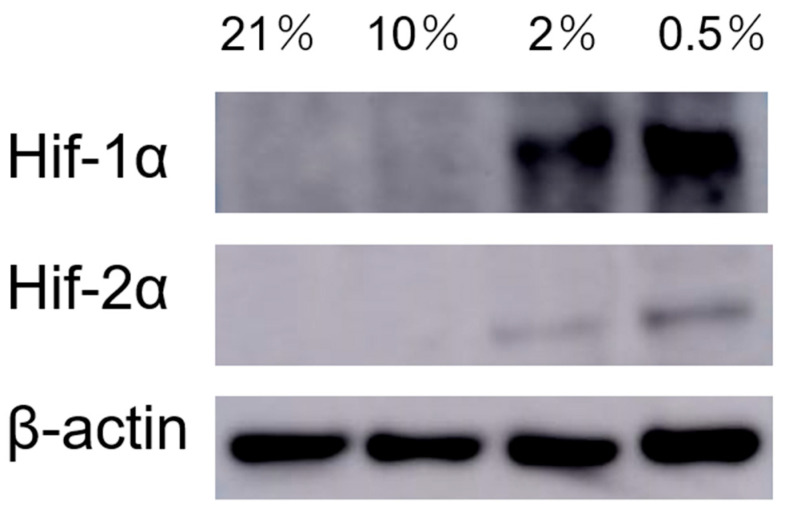
Protein expression of HIF-1α and HIF-2α in sweat gland spheroids cultured under various O_2_ conditions. β-actin is shown as an endogenous control (*n* = 4).

Next, to investigate the oxygen concentrations under which sweat gland cells express HIF-1α and HIF-2α in vitro, we evaluated protein expression in primary cultured human sweat gland cell spheroids that were cultured for 2 weeks under 0.5%, 2%, 10%, and 21% O_2_ conditions. We set O_2_ concentrations to 10% as the highest in vivo O_2_ concentration, 2% as the O_2_ concentration in the epidermis, and 0.5% as the O_2_ concentration in the skin appendages [9,14]. HIF-1α and HIF-2α were expressed in cells cultured under 2% and 0.5% O_2_ conditions and were higher at 0.5% compared with other conditions (Figure 3).

### 2.2. Hypoxic Culture Enhances Myoepithelial Cell Marker Expression as Well as Stem Cell Maintenance-Associated Markers in Sweat Gland Cells

**Figure 4:**
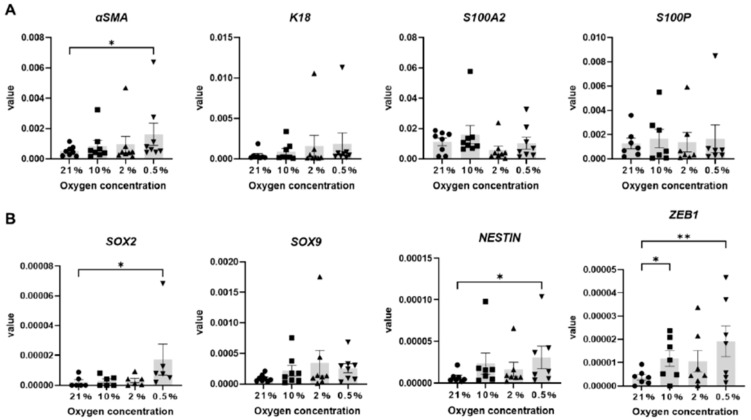
Expression of (**A**) sweat gland cell markers and (**B**) markers important for stem cell maintenance in low-O_2_ conditions and 21% O_2_. Data are shown ± SEM. * *p* < 0.05, ** *p* < 0.01, Friedman test and Dunn’s multiple comparisons test (*n* = 6–8).

To clarify the effect of hypoxia on sweat gland cells cultured in vitro, mRNA expression levels of sweat gland cell markers and markers important for stem cell maintenance in sweat gland cell spheroids were confirmed using real-time quantitative polymerase chain reaction (RT-qPCR). The mRNA expression of *αSMA*, a marker of myoepithelial cells with stem-like cell characteristics in the secretory duct, was significantly higher in the 0.5% O_2_ condition than that in the 21% O_2_ condition (Figure 4A). In contrast, the expression levels of K18, markers for luminal cells in the secretory duct; S100A2, markers for excretory basal layer cells; and S100P, markers for excretory luminal cells were not significantly altered (Figure 4A). In addition, the mRNA expression levels of *SOX2* (SRY-Box Transcription Factor 2), *NESTIN*, and *ZEB1* (Zinc Finger E-Box Binding Homeobox 1) in the 0.5% O_2_ condition were also significantly higher than those in the 21% O_2_ condition (Figure 4B).

**Figure 5:**
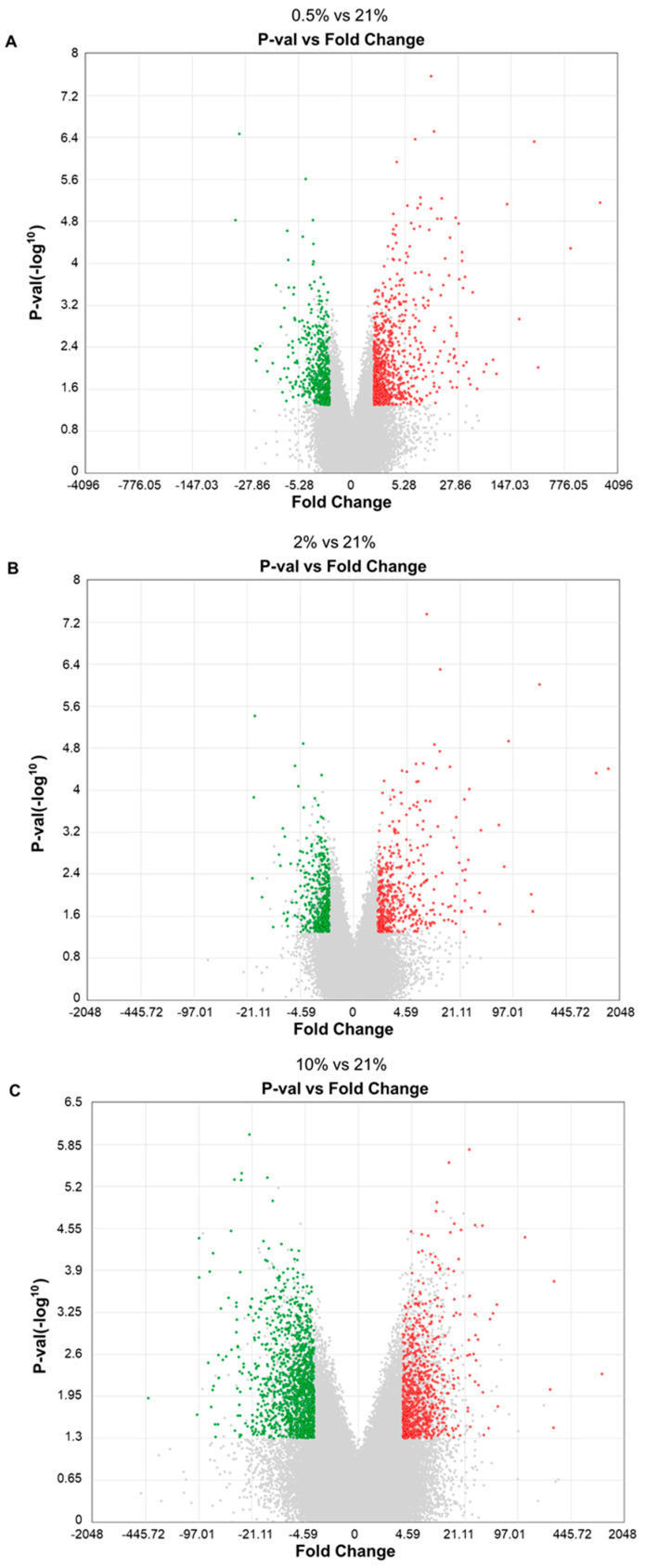
Genes that fluctuated in sweat gland cells cultured in hypoxia by DNA microarray (*n* = 3). Green dots indicate genes significantly decreased less than 0.5-fold, and red dots indicate genes significantly increased by more than 2-fold. (**A**) Expression levels of 778 genes increased, and 526 genes decreased in the 0.5% O_2_ condition. (**B**) Expression levels of 476 genes increased, and 457 genes decreased in the 2% O_2_ condition. (**C**) Expression levels of 831 genes increased, and those of 1282 genes decreased in the 10% O_2_ condition.

**Figure 6:**
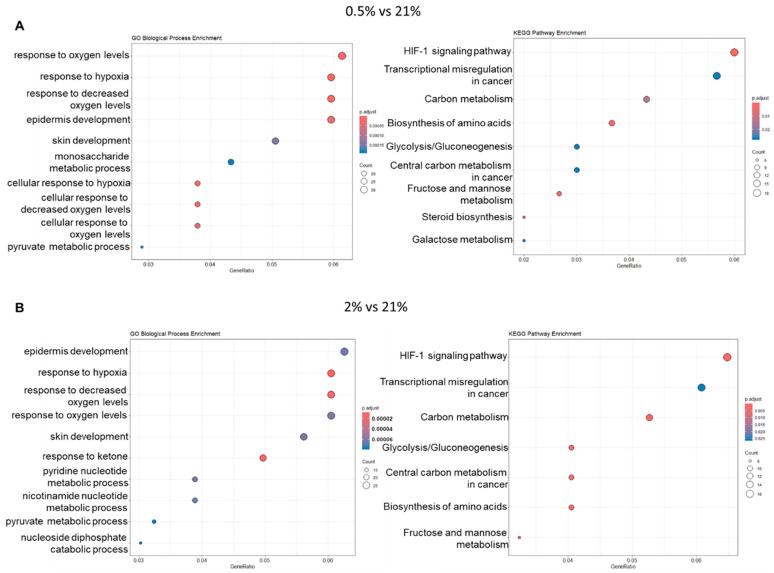
Gene expression analysis in sweat gland cells cultured in hypoxia (*n* = 3). (**A**) GO and KEGG enrichment analysis of genes with increased and decreased expression in the 0.5% O_2_ condition. (**B**) Go and KEGG enrichment analysis of genes with increased and decreased expression in the 2% O_2_ condition.

A cDNA microarray was performed to elucidate how hypoxia modulates the properties and physiological activity of sweat gland cells cultured in vitro. Genes that fluctuated under hypoxia (*p* < 0.05, fold change: <−2 or >2) compared to the 21% O_2_ condition were extracted. The total number of genes whose expression level changed in the 0.5% O_2_ condition was 1304, including 778 genes with increased expression and 526 genes with decreased expression (Figure 5A). In the 2% O_2_ condition, a total of 933 genes were altered, including 476 with increased expression and 457 with decreased expression (Figure 5B). In the 10% O_2_ condition, a total of 2113 genes were altered, including 831 genes with increased expression and 1282 genes with decreased expression (Figure 5C). In addition, Gene Ontology (GO) and Kyoto Encyclopedia of Genes and Genomes (KEGG) pathway enrichment analysis was performed using the fluctuating genes; genes that fluctuated in the 0.5% O_2_ condition showed enrichment in biological processes and transduction pathways related to hypoxia response, epithelial and skin formation, and metabolism (Figure 6A). The enrichment analysis results in the 2% O_2_ condition were similar to those in the 0.5% O_2_ condition (Figure 6B). However, no significant GO enrichment results were identified in the 10% O_2_ condition because of the abundant number of genes that had variable expression. If a large number of genes are significantly altered, this may also affect the background gene set used for comparison, potentially reducing the statistical power of the analysis. If the majority of genes in the background set are also significantly altered, this may cause enrichment for a particular GO term to go undetected [16]. Therefore, GO and KEGG pathway enrichment analyses were performed for genes that were up- and down-regulated in the 10% O_2_ condition, respectively. However, only downregulated genes in the 10% O_2_ condition showed significant enrichment for the GO term “inhibition of the apoptotic execution step”.

### 2.3. Hypoxic Culture Potentially Activates Wnt and TGFβ1 Signaling Pathways

We focused on the 0.5% O_2_ condition because it was the only condition that showed a significant increase in mRNA expression of *αSMA* and markers important for stem cell maintenance compared to the 21% O_2_ condition, even though the gene expression profiling results for the 0.5% and 2% O_2_ conditions were comparable.

Wnt signaling regulates the HIF-1 signaling pathway and ESG development. TGFβ1 signaling also triggers diverse cellular responses and plays important roles in embryonic development, especially in mammary gland development, similar to sweat glands, wound healing, and tissue homeostasis. To validate the microarray results, changes in genes involved in Wnt signaling and TGFβ1 signaling, in addition to hypoxia-related genes, were followed by RT-qPCR analysis.

**Figure 7:**
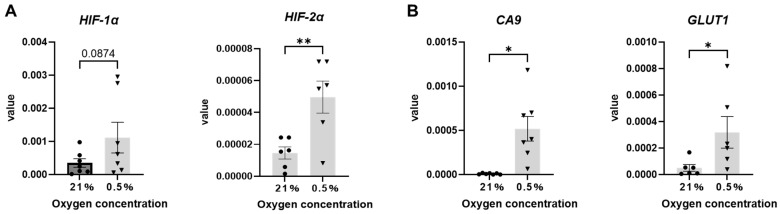
Expression levels of genes or proteins involved in hypoxic response. Data are shown ± SEM. * *p* < 0.05, ** *p* < 0.01, paired *t*-test. (*n* = 6–7) (**A**) *HIF-1α* and *HIF-2α* (**B**) *CA9* and *GLUT1* mRNA expression in the 0.5% vs. 21% O_2_ condition.

**Figure 8:**
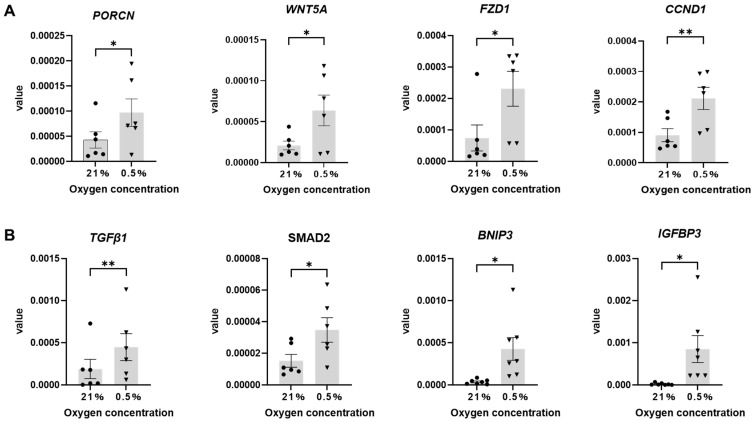
Expression levels of genes involved in Wnt signaling and TGFβ1 signaling. Data are shown ± SEM. * *p* < 0.05, ** *p* < 0.01, paired *t*-test. (**A**) mRNA expression of PORCN, WNT5A, FZD1 and CCND1, genes related to the Wnt signaling pathway (*n* = 6). (**B**) mRNA expression of TGFβ1, SMAD2, IGFBP3, and BNIP3, genes related to the TGFβ1 signaling pathway (*n* = 6–7).

The mRNA expression of *HIF-1α*, the most important factor in the HIF-1 signaling pathway, showed an increasing trend in the 0.5% O_2_ condition (Figure 7A). *HIF-2α*, *CA9* (Carbonic anhydrase IX) [17,18], and *GLUT1* (Glucose transporter 1) [19,20] are markers of hypoxia that are also involved in metabolic pathways. The mRNA expression levels of these three genes were significantly increased in the hypoxia condition compared to that in the 21% O_2_ condition (Figure 7B). Compared to the 21% O_2_ condition, mRNA expression levels of *PORCN* (Porcupine O-Acyltransferase), *WNT5a*, the receptor *FZD1* (Frizzled Class Receptor 1), and the downstream factor *CCND1* (Cyclin D1) [21], which are involved in Wnt protein secretion in the 0.5% O_2_ condition, were significantly increased (Figure 8A).

The mRNA expression levels of *TGFβ1*, *SMAD2* (SMAD Family Member 2), *IGFBP3* (Insulin-Like Growth Factor Binding Protein 3), and *BNIP3* (BCL2 Interacting Protein 3) were also significantly increased (Figure 8B). Furthermore, to clarify the involvement of TGFβ1 and Wnt signaling in hypoxic culture, changes in pSMAD2/3 and β-Catenin expression at the protein level were confirmed using Western blotting and immunostaining (Supplemental Figure S1A,B). The results showed an increase in pSMAD2/3 and β-Catenin expression in the 0.5% O_2_ condition as compared to the 21% O_2_ condition. These results indicated TGFβ1 and Wnt signaling pathway upregulation. The Western blot also revealed an increase in the expression level of αSMA, a marker of sweat gland myoepithelial cells (Supplemental Figure S1A).

## 3. Discussion

Stemness maintenance and differentiation induction at the appropriate time in culture is important for in vitro model building [22]. Sweat gland cell differentiation is induced in conventional sweat gland cell monolayer culture, promoting differentiation into keratinocytes and maintaining sweat gland-specific phenotypic and functional characteristics [23,24]. Although cell-directed differentiation can be induced in three-dimensional (3D) culture conditions, studies regarding stemness maintenance in sweat gland cells remain limited [25,26]. In this study, we focused on the stem cell niche and explored the effects of hypoxic microenvironments on sweat gland cells and their regulatory mechanisms in long-term ex vivo culture.

HIF-1α expression in secretory and excretory ducts of sweat glands and HIF-2α expression in secretory ducts in human skin tissue validated by immunostaining suggested that sweat glands possibly have a hypoxic microenvironment in vivo. In particular, HIF-2α was expressed only in myoepithelial cells in the secretory portion and secretory cells (clear cells). Clear cells have intercellular tubules; contain glycogen; have large amounts of mitochondria and high Na^+^/K^+^-ATPase activity; and are responsible for primary sweat secretion, which is nearly isotonic to plasma. Myoepithelial cells function as a secretory efflux mechanism in the sweat gland through coordinated contractions via gap junctions [27]. Thus, HIF-2α may potentially be involved in the secretory function of sweat gland secretory ducts; however, its specific role remains unelucidated. HIF-1α and HIF-2α expression in myoepithelial cells, which are progenitor cells, suggests that they could maintain an undifferentiated state. HIF-1α and HIF-2α protein expressions in spheroid-cultured sweat gland cells were strong at 0.5% and weak at 2%, suggesting that sweat gland cells could respond to hypoxia at relatively low oxygen concentrations.

Gene expression analysis also revealed significantly increased expression of *αSMA*, a marker specific for myoepithelial cells, in sweat gland cell spheroids in hypoxic cultures. Although the expression and role of *NESTIN*, *ZEB1*, and *SOX2*—which were higher in the 0.5% O_2_ condition than in the other condition—have not been explored in the sweat gland, studies have reported that these genes are essential for maintaining the undifferentiated properties of epidermis and hair follicle cells and for regulating their proliferation [28,29,30]. Taken together, hypoxic environments might maintain the stem cell-like properties of sweat gland cells cultured in vitro.

GO and KEGG pathway enrichment analysis revealed that genes significantly altered in the 0.5% and 2% O_2_ conditions were enriched in HIF-1 signaling or epithelial and skin formation, as well as in biological processes and metabolism-related pathways. We focused on Wnt/β-Catenin and TGFβ1 signaling, which are involved in gland organ development and HIF-1 signaling [31,32,33,34]. Activation of Wnt/β-Catenin maintains the self-renewal capacity and pluripotency of embryonic stem cells in human and murine models [35]. This activation in turn promotes the expression of genes associated with pluripotency by stabilizing β-Catenin, which activates transcription factors such as TCF/LEF [36]. Hypoxic environments promote β-Catenin stabilization and translocation to the cell nucleus via a hypoxia-inducible factor-dependent mechanism [33]. HIF-2α also activates β-Catenin in hypoxic environments [37]. PORCN is a membrane-bound O-acyl transferase essential for all Wnt ligands’ transport, secretion, and activity. When PORCN is deficient, cells cannot secrete functional Wnt ligands, and Wnt signaling by autocrine and paracrine pathways is wholly abolished [38,39]. Gene expression analysis revealed a significant increase in *PORCN* expression in sweat gland cells cultured under hypoxic conditions. Simultaneously, gene expression levels of the Wnt ligand *WNT5A* and Wnt receptor *FZD1* were also significantly increased. Although Wnt5a does not directly upregulate Wnt/β-Catenin signaling, it can activate Wnt/β-Catenin signaling in cooperation with other biological processes [40,41]. Furthermore, genetic defects in *WNT5A* have been reported to suppress Wnt/β-Catenin signaling [42]. In contrast, *FZD1* mediates Wnt/β-Catenin signal transduction via direct ligand interaction [43]. *FZD1* upregulation causes sustained Wnt/β-Catenin signaling activation, resulting in β-Catenin translocation into the cell nucleus and enhanced transcriptional activation of target genes [44]. Increased *CCND1* expression, a known transcript of Wnt/β-Catenin signaling, suggests that the hypoxic environment could enhance Wnt/β-Catenin signaling.

TGFβ1 signaling plays a complex role in maintaining and regulating stemness. The TGFβ1 signaling ligand and its important downstream mediator *SMAD2* was significantly upregulated in sweat gland cells under hypoxic culture conditions. HIF-1α, HIF-2α, and TGFβ1/SMAD regulate their signaling activity and gene expression [34,45,46,47]. Activation of TGFβ1/SMAD signaling has been reported to induce expression of the stemness factors *SOX2* and *SOX4*. BNIP3 and IGFBP3, which were significantly upregulated in hypoxic conditions, are downstream factors of TGFβ1 signaling. *IGFBP3* and *BNIP3* play complex roles in regulating proliferation, stemness maintenance, and apoptosis [48,49,50]. *BNIP3* regulates mitochondrial function and lipid synthesis [51,52]. IGFBP3 is also involved in sugar and lipid metabolism and suppresses mesenchymal stem cell differentiation in hypoxic environments [53,54]. These findings suggest that TGFβ1 signaling could cooperate with HIF-1 signaling, which is activated by HIF-1α and HIF2α proteins, to regulate the metabolism and physiological activity of sweat gland cells cultured in hypoxic conditions.

The maintenance of sweat gland stemness not only plays a role in constructing research models but is also closely related to repairing damaged skin [23]. The results of this study provide a new perspective for the long-term culture of sweat gland cells while maintaining their myoepithelial cell-like characteristics. We acknowledge the limitations of this study. First, the involvement of HIF-1, Wnt/β-Catenin, and TGFβ1 signaling pathways in stem cell maintenance remains to be further elucidated through molecular and cellular analyses, including ex vivo and in vivo studies, to better understand multicellular and systemic physiological interactions. Second, the evaluation of physiological sweat gland functions (including water channel activation) and the functional analysis of sweat gland regenerative capacity are important issues for future investigation. Furthermore, to assess the potential for clinical application of hypoxia-cultured sweat glands, it will be necessary to conduct long-term in vitro cultures and subsequent in vivo transplantation experiments using models of damage or disease to evaluate regenerative ability and function. Another limitation of this study is the relatively small sample size, which may reduce statistical power and limit the generalizability of the findings. In the future, we will extend the findings of this study to research on sweat gland function, the development of therapeutic agents for sweating disorders, and regenerative medicine for sweat glands.

## 4. Materials and Methods

### 4.1. Ethics Statement

All procedures performed in studies involving human participants followed the ethical standards of the institutional and/or national research committee and were in accordance with the 1964 Helsinki Declaration and its later amendments or comparable ethical standards. The protocol for obtaining skin samples was approved by the Ethics Committee of the University of Osaka (yakuhito 2024-14, yakuhito 28-3, yakuhito 2019-28). Fresh human postoperative skin specimens were obtained with informed consent from all participants at the Plastic Surgery Department at the University of Osaka Hospital (Osaka, Japan), Kinugasa Clinic (Osaka, Japan), and the Seishin Plastic and Aesthetic Surgery Clinic Co., Ltd. (Tokyo, Japan). The average age of the participants that the specimens were obtained from was 47.35 ± 9.13 years.

### 4.2. Primary Sweat Gland Cell Spheroid Culture

We followed the procedure of Hayakawa et al. for cell culturing, with some modifications [55]. Briefly, human skin tissue was homogenized and digested with 600 units/mL collagenase type II (Worthington Biochemical Corporation, CLS2, Lakewood, NJ, USA) and 3% penicillin–streptomycin–amphotericin B suspension (Fujifilm, 161-23181, Tokyo, Japan) in Mammocult Human Medium (STEMCELL Technologies, 05620, Vancouver, BC, Canada) supplemented with 10% (*v*/*v*) of MammoCult™ Proliferation Supplement (Human) (STEMCELL Technologies, 05622), 10 ng/mL of Animal-Free Recombinant Human EGF (PeproTech, AF-00-15, Cranbury, NJ, USA), 10 ng/mL of Human Recombinant bFGF (STEMCELL Technologies, 78003), 4 µg/mL of heparin solution (STEMCELL Technologies, 07980), 0.5 µg/mL of hydrocortisone 21-hemisuccinate (Sigma-Aldrich, H2882-1, St. Louis, MO, USA), and 2% (*v*/*v*) of Matrigel^®^ Growth Factor Reduced (Corning, 3471, Corning, NY, USA) in a 37 °C, 5% CO_2_ incubator for 16 h. Sweat glands were removed from the digested skin tissue with a micropipette and washed with phosphate-buffered saline (PBS); then, 3 mL of Accutase (BD Biosciences, 561527, Franklin Lakes, NJ, USA) was added and incubated at 37 °C for 20 min, and sweat gland cells were sorted into single cells by pipetting.

Sweat gland cells were seeded in ultra-low-adherence surface 6-well plates (CORNING, 3471) in supplemented Mammocult Human Medium. The control condition was incubated at 21% O_2_, and cells were cultured under 0.5%, 2%, and 10% oxygen conditions in an incubator (Eppendorf, cellxpert c170i, Hamburg, Germany) or a hypoxia incubator chamber (STEMCELL Technologies, ST-27310). The medium was not changed until cell collection. Cells were harvested on day 14 of the culture.

### 4.3. Immunohistochemical Analysis

Human skin tissue was placed in 4% paraformaldehyde phosphate buffer (4% PFA) (Fujifilm, 163-20145) for tissue fixation and fixed overnight at 4 °C. The next day, after washing with PBS, paraffin blocks were prepared by dehydration, organic solvent substitution, and paraffin substitution. Sweat gland spheroids were fixed in 4% PFA and embedded and frozen in Tissue-Tek^®^ O.C.T. Compound (Sakura Fine tek, Tokyo, Japan). Paraffin blocks and frozen blocks were cut to a thickness of 5 µm. Paraffin sections were deparaffinized and incubated in Tris-EDTA buffer at 100 °C for 20 min for activation. Both paraffin and frozen sections were incubated with 5% bovine serum albumin (BSA) (Sigma, A7030)/PBS for 1 h at room temperature for blocking; this was followed by primary antibody reaction by diluting the antibodies according to the dilution ratio of each antibody in 2% BSA/PBS/0.1% tween 20 (Sigma, SLCJ0231) and incubating overnight at 4 °C. The primary antibodies used and dilution factors were HIF-1α (SantaCruz, sc-53546, 1:100, Dallas, TX, USA), HIF-2α (Abcam, ab109616, 1:100), S100A2 (Abcam, ab109494, 1:100, Cambridge, UK), S100A2 (ABCEPTA, AM8507b, 1:25, San Diego, CA, USA), αSMA (Abcam, ab5694, 1:100), FOXA1 (Abcam, ab55178, 1:200), AQP5-AF488 (SantaCruz, sc-514022, 1:500), αSMA-APC (R&D, IC1420A, 1:50, Minneapolis, MN, USA), and β-Catenin (Cell Signaling Technology, 37447, 1:400, Danvers, MA, USA). The following day, after washing with PBS, the following secondary antibodies were added: Anti-mouse IgG, Alexa Fluor™ 488 (Thermo Fisher Scientific, A-11029, Waltham, MA 02451 USA), Alexa Fluor™ 594 (Thermo Fisher Scientific, A-21203), Anti-rabbit IgG, Alexa Fluor™ 594 (Thermo Fisher Scientific, A-21207), Alexa Fluor™ 488 (Thermo Fisher Scientific, A-21206), Anti-mouse IgG Nano (VHH), Alexa Fluor™ 568 (Jackson ImmunoResearch, SA5-10325, West Grove, PA, USA), and Alexa Fluor™ 488 (Jackson ImmunoResearch, 615-544-214). These were adjusted to 1:500 for VHH antibodies and 1:200 for the others with 2% BSA/PBS/0.1% tween 20. Then, Hoechst 33342 (Thermo Fisher Scientific, H3570) diluted to 1:1000 was added and incubated at room temperature for 2 h. After washing, the cells were sealed in a covered glass using Prolong Gold encapsulant (Thermo Fisher Scientific, P36980) and observed with a confocal laser scanning microscope (Evident Scientific, FV3000, Tokyo, Japan).

### 4.4. RNA Extraction

Sweat gland spheroids were collected and processed using NucleoSpin^®^ RNA Plus (MACHEREY-NAGEL, 740984, Düren, Germany), according to the manufacturer’s instructions, to obtain RNA samples.

### 4.5. DNA Microarray

DNA microarrays (Clariom™ D Array, human, Thermo Fisher Scientific, 902915) were performed according to the manufacturer’s protocol. Extracted RNA was amplified, labeled, and purified using the GeneChip^®^ WT PLUS Reagent Kit (Thermo Fisher Scientific, 902280). Hybridization and washing before arrays were performed using the GeneChip^®^ Hybridization, Wash and Stain Kit (Thermo Fisher Scientific, 900720); Hybridization Oven 645; and Fluidics Station 450 (both processes were performed by Affymetrix, and arrays were scanned on a GeneChip^®^ Scanner 3000 (Thermo Fisher Scientific).

Data analysis was performed using Transcriptome Analysis Console software ver 4.0.3.14 (Thermo Fisher Scientific) and clusterProfiler ver 4.12.0 of the R Package.

### 4.6. RT-qPCR

Extracted RNA was used to synthesize cDNA using the QuantiTect Reverse Transcription Kit (Qiagen, 205313, Hilden, Germany), and the PCR reaction solution was prepared using the THUNDERBIRD Next SYBR qPCR Mix (TOYOBO, QPX-201, Osaka, Japan). Gene expression levels were analyzed using the QuantStudio™ 6 Pro Real-Time PCR System (Thermo Fisher), and 40 cycles were performed at 95 °C for 15 s; 55 °C for 10 s; and 72 °C for 30 s. Subsequently, 18s RNA was used as an endogenous control for detection, and relative expression levels were calculated. The primers used for genetic analysis are listed in Table 1.

### 4.7. Western Blotting

Sweat gland spheroids were dissolved in RIPA buffer (50 mM Tris-HCI; pH 7.5, 150 mM NaCl, 1% NP-40, 0.5% sodium deoxycholate, 0.1% SDS), mixed with 4× Laemmli sample buffer (Biorad, 1610747, Hercules, CA, USA), and heated at 95 °C for 5 min. Samples were loaded into wells of TGX™ FastCast™ acrylamide gels and transferred to a polyvinylidene fluoride (PVDF) membrane (Biorad, 1704156) using the TransBlot Turbo™ transcription system (Biorad, 1704150). The membranes were immersed in primary antibodies diluted in 5% BSA or 5% skim milk in Tris-buffered saline containing Tween-20 (TBST) following the instruction of the data sheet and incubated at 4 °C overnight. The primary antibodies used were HIF-1α (Cell Signaling Technology, 36169), HIF-2α (Cell Signaling Technology, 7096), αSMA (Abcam, ab5694), β-Catenin Cell Signaling Technology, 37447), Phospho-SMAD2 (Ser465/467)/SMAD3 (Ser423/425) (Cell Signaling Technology, 8828), SMAD2/3 (Cell Signaling Technology, 8685), and β-actin (Cell Signaling Technology, 4970). After three washes with TBST, the membrane was incubated with 5% skim milk; diluted Anti-rabbit IgG, HRP-linked (1: 2000, Cell Signaling Technology, 7074S); or Anti-mouse IgG, HRP-linked (1:2000, Abcam, ab6823) and incubated at room temperature for 1 h. After the antibody reaction, the membrane was washed three times in TBST and then reacted with ECL solution (Cytiva, RPN2236, Marlborough, MA, USA) for 5 min on the membrane. Image acquisition was performed using an Amersham Imager 600 (Cytiva).

### 4.8. Statistical Analysis

GraphPad Prism ver 9.5.1 (GraphPad Software, San Diego, CA, USA) was used for the statistical analysis. Data were obtained from at least six independent experiments and tested for significant differences using Friedman and Dunn’s multiple comparisons tests in multi-group data. Paired *t*-tests were also performed in the case of two groups. *p* values were set as follows: * *p* < 0.05, ** *p* < 0.01, and *** *p* < 0.001.

## Figures and Tables

**Table 1:** Sequence of primers for RT-qPCR.

Gene	Forward (5′ to 3′)	Reverse (5′ to 3′)
*18S*	CGGCTACCACATCCAAGGAA	AGCTGGAATTACCGCGGC
*HIF*-*1*α	TTAGAACCAAATCCAGAGTCAC	TATTCACTGGGACTATTAGGCT
*HIF2*α	ACCTGAAGATTGAAGTGATTGAG	GTGGCTGGAAGATGTTTGTC
*CA9*	TTTGCCAGAGTTGACGAGGC	GCTCATAGGCACTGTTTTCTTCC
*α* *SMA*	ATAGAACATGGCATCATCACCAAC	GGGCAACACGAAGCTCATTGTA
*Keratin 18*	CCCTGCTGAACATCAACCTCAA	GCTGTCCAAGGCATCACCAA
*S100A2*	GCCAAGAGGGCGACAAGTT	AGGAAAACAGCATACTCCTGGA
*S100P*	AAGGATGCCGTGGATAAATGC	ACACGATGAACTCACTGAACTC
*PORCN*	CCCCTCCATCCTTGACC	CTCCCCTTCTCTGTTTCCC
*W* *NT* *5* *A*	CCTGAAGGAGAAGTACGACAG	GATGTAGACCAGGTCTTGTGTG
*FZD1*	ACTCCCTTCTCCCACCTTAGTT	ATGCTTCTTCCCAAATCTCAGT
*CCND1*	AGAGGCGGAGGAGAACAAAC	AAGCGTGTGAGGCGGTAGTA
*TGF* *β* *1*	ACAATTCCTGGCGATACCTCAGCA	TCTTCTCCGTGGAGCTGAAGCAAT
*SMAD2*	GCCTTTCAGCTTCTCTGAACAA	ATGTGGCAATCCTTTTCGAT
*IGFBP3*	GCTCTGCGTCAACGCTAGTG	GCTTCCTGCCTTTGGAAGGG
*BNIP3*	CAGGGCTCCTGGGTAGAACT	CTACTCCGTCCAGACTCATGC
*SOX2*	CCCCCGGCGGCAATAGCA	TCGGCGCCGGGGAGATACAT
*SOX9*	AGCGAACGCACATCAAGAC	CTGTAGGCGATCTGTTGGGG
*NESTIN*	CTGCTACCCTTGAGACACCTG	GGGCTCTGATCTCTGCATCTAC
*ZEB1*	CAGCTTGATACCTGTGAATGGG	TATCTGTGGTCGTGTGGGACT
*G* *LUT1*	CTGCAACGGCTTAGACTTCGAC	TCTCTGGGTAACAGGGATCAAACA

## Data Availability

Data is contained within the article and Supplementary Materials.

## Supplementary Materials

The following supporting information can be downloaded at: https://www.mdpi.com/article/10.3390/ijms26146664/s1.
