# Supplementary material for: Long-Term Hypoxia Upregulates Wnt and TGFβ1 Signaling in Eccrine Sweat Gland Cells In Vitro
Source: Int J Mol Sci. 2025 Jul 11;26(14):6664. doi: 10.3390/ijms26146664 (PMC12294486; doi:10.3390/ijms26146664)
Supplement: Supplementary file 1 [file ijms-26-06664-s001.zip › ijms-3725607-supplementary.pdf]

Supplemental Figure S1

A

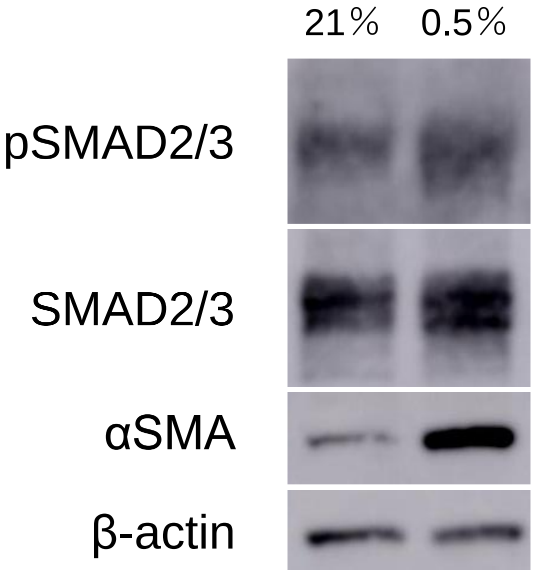


B

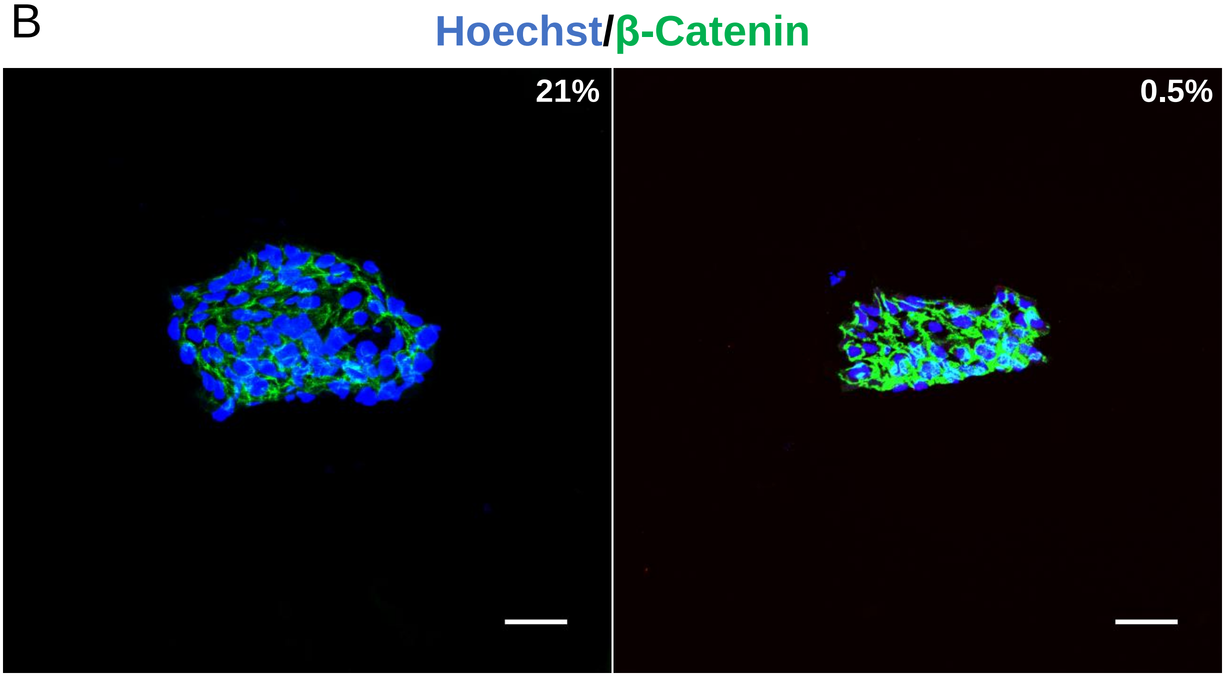


Supplemental Figure S1
(A)Protein expression of pSMAD2/3, SMAD2/3 and αSMA. β-actin is shown as an endogenous control (n = 4). (B)Expression of β-Catenin in sweat gland spheroids. Blue indicates nuclei in Hoechst, green indicates β-Catenin Scale bar 30 µm (n = 3).
